# Evaluating the utility of deep learning for predicting therapeutic response in diabetic eye disease

**DOI:** 10.3389/fopht.2022.852107

**Published:** 2022-08-12

**Authors:** Vincent Dong, Duriye Damla Sevgi, Sudeshna Sil Kar, Sunil K. Srivastava, Justis P. Ehlers, Anant Madabhushi

**Affiliations:** 1 The Center for Computational Imaging and Personalized Diagnostics, Case Western Reserve University, Department of Biomedical Engineering, Cleveland, OH, United States,; 2 The Tony and Leona Campane Center for Excellence in Image-Guided Surgery and Advanced Imaging Research, Cleveland Clinic, Cleveland, OH, United States,; 3 Wallace H Coulter Department of Biomedical Engineering, Emory University and Georgia Institute for Technology, Atlanta, GA, United States,; 4 Atlanta Veterans Administration Medical Center, Atlanta, GA, United States

**Keywords:** deep learning, diabetic retinopathy, diabetic macular edema, ultra-widefield fluorescein angiography, optical coherence tomography, transfer learning

## Abstract

**Purpose:**

Deep learning (DL) is a technique explored within ophthalmology that requires large datasets to distinguish feature representations with high diagnostic performance. There is a need for developing DL approaches to predict therapeutic response, but completed clinical trial datasets are limited in size. Predicting treatment response is more complex than disease diagnosis, where hallmarks of treatment response are subtle. This study seeks to understand the utility of DL for clinical problems in ophthalmology such as predicting treatment response and where large sample sizes for model training are not available.

**Materials and Methods:**

Four DL architectures were trained using cross-validated transfer learning to classify ultra-widefield angiograms (UWFA) and fluid-compartmentalized optical coherence tomography (OCT) images from a completed clinical trial (PERMEATE) dataset (n=29) as tolerating or requiring extended interval Anti-VEGF dosing. UWFA images (n=217) from the Anti-VEGF study were divided into five increasingly larger subsets to evaluate the influence of dataset size on performance. Class activation maps (CAMs) were generated to identify regions of model attention.

**Results:**

The best performing DL model had a mean AUC of 0.507 ± 0.042 on UWFA images, and highest observed AUC of 0.503 for fluid-compartmentalized OCT images. DL had a best performing AUC of 0.634 when dataset size was incrementally increased. Resulting CAMs show inconsistent regions of interest.

**Conclusions:**

This study demonstrated the limitations of DL for predicting therapeutic response when large datasets were not available for model training. Our findings suggest the need for hand-crafted approaches for complex and data scarce prediction problems in ophthalmology.

## 1 Introduction

**FIGURE 1:**
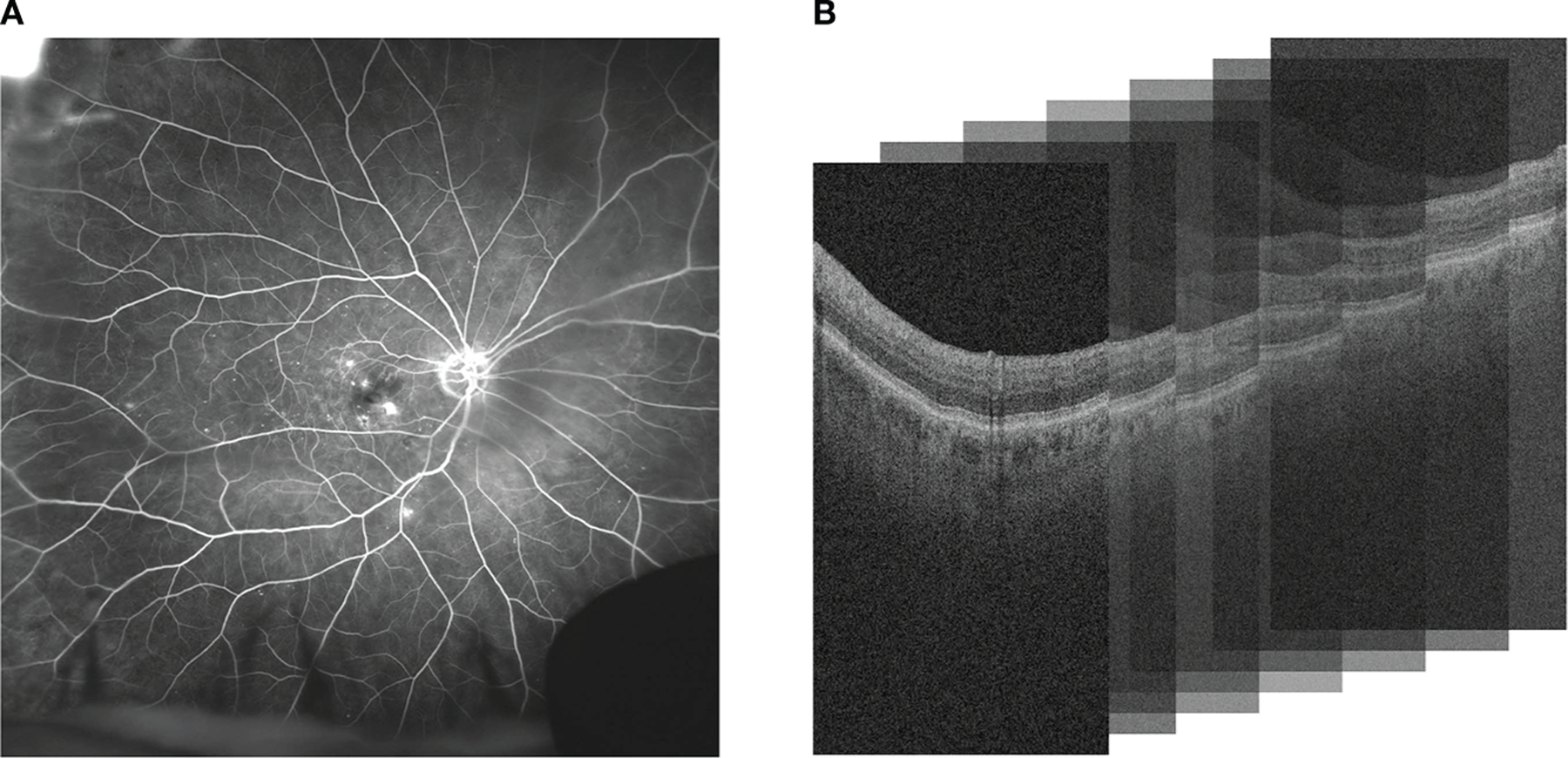
Comparison of **(A)** UWFA and **(B)** OCT imaging modalities. UWFA is a wide angle scan of the retinal surface and OCT is a volumetric scan of the retina that can be decomposed into 2D OCT B-scans. Note that in **(B)**, the 3D volume is represented as a stack of the 2D B-scans used in this study.

Diabetic retinopathy (DR) is the most common cause of vision loss and blindness in working-age adults. DR is thought to be present in approximately 30% of diabetic individuals over the age of 40 in the United States (1). Diabetic macular edema (DME), a serious complication of DR, is intracellular fluid accumulation within the macular region. Retinal vein occlusion (RVO) is the second most common retinal vascular disease and can result in visually-significant macular edema (2). Vascular Endothelial Growth Factor (VEGF) has been recognized as an important cytokine that induces retinal vascular hyperpermeability in both DME and RVO. This results in the accumulation of intraretinal fluid (IRF) or subretinal fluid (SRF) in the retina. Anti-VEGF is the current standard treatment in DME and RVO to resolve leakage accumulation. The response to anti-VEGF can be variable and interval tolerance between treatments is not currently predictable based on traditional clinical features. Ultra-widefield fluorescein angiography (UWFA) and Optical coherence tomography (OCT) are standard imaging modalities used for diagnostics of patients with DME or RVO. UWFA enables analysis of pan retinal vascular abnormalities, such as fluid leakage, micro-aneurysms and non-perfusion. OCT allows detailed anatomic evaluation of retinal layers (3). UWFA and OCT imaging modalities are illustrated in Figure 1.

Deep learning (DL) is a class of machine learning that uses configurations of deep neural networks using unsupervised feature generation techniques to distinguish categories of interests (4–7). DL has been employed to great effect in many application areas within ophthalmology, mostly revolving around disease detection and diagnosis. Different modalities of data also impact the performance of DL models, as visual markers are captured and presented differently depending on the imaging modality (8). Both UWFA and OCT modalities have been used in DL-based experiments for diagnosis related tasks and have achieved robust classification performance on large datasets (4–7).

DL approaches have also been used for detection and segmentation of specific hallmarks of disease such as the approach by Schlegl et al. where DL approaches were employed for quantifying macular fluid in OCT images (n = 1200), yielding a mean AUC of 0.94 (4). Lee et al. used DL to distinguish normal OCT images versus age-related macular degeneration images (n = 101,002), yielding an AUC of 0.93 (5). Varadarajan et al. predicted DME grades from fundus photos (n = 7072), yielding an AUC of 0.89 (6). Different DL model architectures were used for these aforementioned studies (4–6) where imaging datasets had sizes greater than a thousand samples available for training. In these previously mentioned disease classification studies where identifiers for disease presence are quite discernible, DL models were provided with training sets upwards of thousands of samples (4–6). When presented with large amounts of data for a task that contains obvious hallmarks of disease presence, DL-based approaches yield good classification results (4–6). In disease diagnosis tasks, visual markers have been identified in OCT or fundus photos. Specifically for diagnosing DR, similar works have utilized visual markers from various retina modalities to high levels of success (9, 10). Sandhu et al. utilized extracted clinical biomarker features from both OCT and OCT angiography (OCTA) to train a classifier to diagnose and grade the severity of nonproliferative DR, classifying samples as either no DR, mild nonproliferative DR, or moderate nonproliferative DR. Using this classifier, Sandhu et al. reported an AUC of 0.987 when combining features extracted from both modalities (9). Sharafeldeen et al. extracted high-order morphological and novel reflectivity markers per individual segmented retinal layers to detect early DR with OCT B-scans, classifying samples as either normal eyes or eyes containing DR. Each extracted descriptor per retinal layer is fused to create a novel classification model that reached a classification AUC of 0.982 (10).

However, when it comes to a task like predicting treatment response, there are limited hallmarks of disease aggressiveness, or tolerance to treatment. Even clinical interpretation of severity of DME and response to anti-VEGF therapy is highly variable, and subject to the impression of the individual ophthalmologist reviewing the images. Furthermore, the response to therapy can be the same between two patients, but have vastly different late phase image scans due to differences in the initial severity of the disease. Thus, the task of predicting treatment response is potentially more challenging to disease diagnosis. Additionally, in the absence of obvious hallmarks of disease treatment response, unsupervised feature generation approaches like DL could be challenged by the limited size of training datasets available for developing treatment response predictors. In addition, certain emerging modalities may be subject to greater limitations in sample size, such as UWFA and OCTA. Unlike for diagnostic decision-making tasks, where typically large sample set sizes are readily available, predictors for treatment response are typically reliant on completed clinical trial datasets which are usually limited in sample size in the context of ophthalmology. In instances where larger datasets are obtainable however, DL techniques have identified and utilized key OCT image-based features correlated to anti-VEGF response and treatment prognosis (11). Using an OCT dataset of 101 samples, Alryalat et al. propose a novel method of utilizing DL-generated segmentation masks to train a DL classification model to predict treatment response. When training the DL classification model on only the OCT image B-slices, the study reports a classification AUC of 0.76. When the generated segmentation masks are applied to the B-slices, the DL model reaches a high classification AUC of 0.81 (11).

In general applications of DL, data scarcity has been addressed through techniques such as transfer learning, where layers of a neural network are first pre-trained on large general image datasets to learn basic edge and object detection (among other basic feature extractors) (12). As a result, this effectively lowers the dataset size requirement, as only the problem-specific features need to be learned from the data. Then, the last layers of the model are trained specifically on the smaller, desired dataset in order to learn the specified problem. In theory, this approach circumvents the need for extensive model training on a large dataset. Nonetheless, with extreme cases of data scarcity and a complex task, transfer learning may still fail, and be unable to reach optimal classification performance. An alternate approach to deep learning, more apt for data scarce environments, is the radiomics or hand-crafted based feature approach category where pre-defined image attributes are used to predict treatment response (13, 14). For instance, Prasanna (13) and Moosavi (14) separately showed that hand-crafted based radiomic features relating to eye vessels and arrangement of leakage patterns were associated with response to anti-VEGF treatment, albeit for relatively small sample sizes (n=28).

When presented with a more complex task, such as predicting therapeutic response in afflicted eyes, it is important to determine the best strategy to utilize artificial intelligence to solve such a problem. Even with limitations, such as small training dataset size, is it possible to augment the training data in such a way that can lead to optimal predictive performance with DL based models? Does the imaging modality used have an effect on whether DL models can converge in such a problem space? Furthermore, how does the dataset size influence the training of a DL model, and what size dataset should be considered as “large enough” for effective training? Finally, is it possible for DL to detect and learn an underlying signal for a task that does not have obvious visual cues, such as identifying whether a patient is a rebounder or non-rebounder based off their imaging scans? This study seeks to preliminarily explore these questions for the task of predicting therapeutic response in ophthalmology images corresponding to two different modalities - OCT and UWFA. To address these questions, we first evaluated the performance of transfer learning on two different imaging modalities, UWFA and OCT, taken from the PERMEATE clinical trial (n = 29), a prospective open-label IRB-approved study that contained eyes with DME and RVO undergoing anti-VEGF therapy (15). Due to small dataset size, leave one out cross validation is used for classifier training and evaluation. To verify our findings, we visualized the areas where the trained DL models identify as categories of interest, through the use of class activation heatmaps, where areas highly influencing classification are highlighted. Separately, the influence of training set size was explored by dividing the larger Anti-VEGF UWFA dataset into smaller subset sizes, where each subset increased by a factor of 28. In summary, this was the first comprehensive attempt to evaluate DL for ophthalmology related treatment response applications, particularly when large amounts of data were not available for training.

## 2 Methodological framework

### 2.1 Description of datasets

#### 2.1.1 PERMEATE dataset

The PERMEATE study is an IRB-approved prospective clinical study established by the Cole Eye Institute at Cleveland Clinic Foundation (CCF) (15). Written consent was obtained to conduct the study. The study aimed to assess the outcomes of Intravitreal Aflibercept Injection (IAI) therapy in eyes with DME or macular edema secondary to RVO. For the first 6 months, subjects receive 2 mg IAI injections every 4 weeks (q4w) and then progress to 8-week dosing periods (q8w) for the remaining 6 months. Inclusion criteria consist of treatment-naive patients ≥ 18 years of age, presence of DME or RVO, and best-corrected visual acuity of 20/25 or worse. UWFA and OCT were obtained at baseline and at specific time points throughout the study. In order to evaluate treatment interval tolerance, eyes were separated into two cohorts based on visual acuity during the first q8w period. Rebounders (n=12) are eyes exhibiting at least 1 letter worsening in best-corrected visual acuity following the initial q8w challenge, and non-rebounders (n=17) are eyes that maintained or improved best-corrected visual acuity. The UWFA PERMEATE data set consists of 29 total samples with the aforementioned class distribution, while the OCT data set observes a dropped rebounder patient from the dataset (due to poor image quality), and thus contains only 28 samples. Patient characteristics for the PERMEATE dataset is shown in Table 1.

#### 2.1.2 Anti-VEGF dataset

The Anti-VEGF dataset consists of UWFA imaging (Optos) from a retrospective image analysis study that was conducted on eyes with DR. The study was approved by the Cleveland Clinical Institutional Review Board. Two-hundred seventeen eyes from 189 patients were included. Eyes are classified as either requiring anti-VEGF treatment (n=141) or not requiring treatment (n=76). Inclusion criteria include eyes with DR of any severity stage, as determined by the International Clinical Diabetic Retinopathy Severity Scale. Exclusion criteria consist of eyes that had undergone panretinal laser photocoagulation at any time or intravitreal pharmacotherapy within the preceding six month time period. Poor UWFA image quality was also a consideration for exclusion, where instances of artifact presence, poor field-of-view, or limited contrast led to the sample being removed from the dataset. This dataset was used to track changes in DL performance for predicting treatment response when training set size is varied. Patient characteristics for the Anti-VEGF dataset is shown in Table 2.

### 2.2 Transfer learning model architectures

Four pre-trained model architectures were used for the classification problem of predicting treatment response in ophthalmology images. These models include ResNet50, ResNet101, Inception-v3 and DenseNet201. The ResNet50 and ResNet101 model architectures utilize stacking of convolutional layers to learn the residuals of the provided input. ResNet50 is a 50 layer residual network, and ResNet101 is a 101 layer residual network (16). The Inception-v3 model increases the depth and width of a deep convolutional network, while keeping the computational budget constant using a sparsely connected architecture (17). DenseNet201 increases the depth of deep convolutional networks through connecting every other layer in the network in a feed-forward framework. The previous feature maps from each layer are used as the next layer’s input, and are propagated throughout the entire model (18). All four of these models are well-established for image classification tasks, and when combined with transfer learning techniques are typically able to converge to an accurate representation of the image data.

Variations of these model architectures have also been previously explored for various ophthalmologic image classification tasks (19–22). Kim and Tran explore several architectures, such as VGG16, VGG19, ResNet50, ResNet152, DenseNet121, and Inception-v3 as feature extractors to develop binary classifiers for categorizing OCT images into Choroidal neovascularization, Diabetic macular edema, Drusen, and Normal patients (19). Khojasteh et al. utilized the ResNet50 model architecture to detect exudates in fundus images (20). Diaz-Pinto et al. employed five different ImageNet pre-trained models (VGG16, VGG19, Inception-v3, ResNet50, and Xception) for automatic glaucoma assessment of fundus images (21). Pan et al. detect and classify lesions of diabetic retinopathy in fundus fluorescein angiography images using DenseNet, ResNet50, and VGG16 model architectures (22). Across a wide variety of image classification tasks, these variations of ImageNet pre-trained model architectures performed optimally on their respective tasks. In our study, four different image classification model architectures were chosen to highlight the sub-optimal classification performance due to limited data, and not to conduct a comprehensive review of every possible network architecture for this image classification task.

### 2.3 Classification methods

Transfer learning classification experiments are performed on both UWFA and OCT scans to classify patients as rebounders or non-rebounders. With small-sized imaging datasets, leave-one-out cross validation (LOOCV) is utilized to maximize the training set size. Through each iteration of LOOCV, one sample is set aside and used for model validation. The rest of the samples are used for training, and this process is repeated with a new model instance for each configuration of a sample being used as the validation sample. This is done to give DL the best chance of learning necessary features from the data. Standard three-fold cross validation was also employed for experiments using the larger dataset. Optimal hyper-parameters (e.g. learning rate, number of epochs) were found for each model through grid-search experiments.

### 2.4 Heatmap visualizations

Class activation maps (CAM) were generated per sample to visually identify the regions of interest (ROI) that the trained model places attention on when making predictions (23). Using the model’s final layer, highly activated pixels were identified and marked with warmer (red) tones. Similarly, pixels of the sample image that have low attention were marked with cooler (blue) tones. The resulting CAMs were analyzed to determine if the ROI’s identified by the model shared correlation with clinically shown identifiers for anti-VEGF treatment response. In addition, the ROIs were compared among samples to verify if the underlying task was being learned by the model, regardless of sample variance.

### 2.5 Implementation details

Transfer Learning models were loaded in through the use of PyTorch, a Python based machine learning framework (24). Through PyTorch, the four aforementioned model architectures were pre-trained on the ImageNet database, with all layer weights frozen except for the final layer. The model training pipeline and CAM generation is also conducted through the PyTorch package.

## 3 Experimental results

### 3.1 Experiment 1: utility of DL on limited UWFA data

Ability for DL to predict treatment response on UWFA imaging data was evaluated on the PERMEATE dataset. 3900×3072 UWFA late-phase frames for 29 eyes are imported. The images are first resized to 508×400 and then center cropped to 224×224 to match input dimensions of the pre-trained DL models. This process also removes the artifacts on the outer regions of the images such as eyelashes and eyelids. Finally, normalization is applied to match ImageNet standards. A patch-based approach and the use of data augmentations were also explored in experiments detailed in Section 1 and 2.1 of the Supplementary Material.

Transfer learning, as described in Section 2, is applied to these processed images and LOOCV is employed due to the small sample size of the PERMEATE dataset (n=29). Training is done over 30 seeded runs, with 100 epochs per training set and a learning rate of 0.001. Then, resulting CAMs are generated per trained model for the set of samples.

**FIGURE 2:**
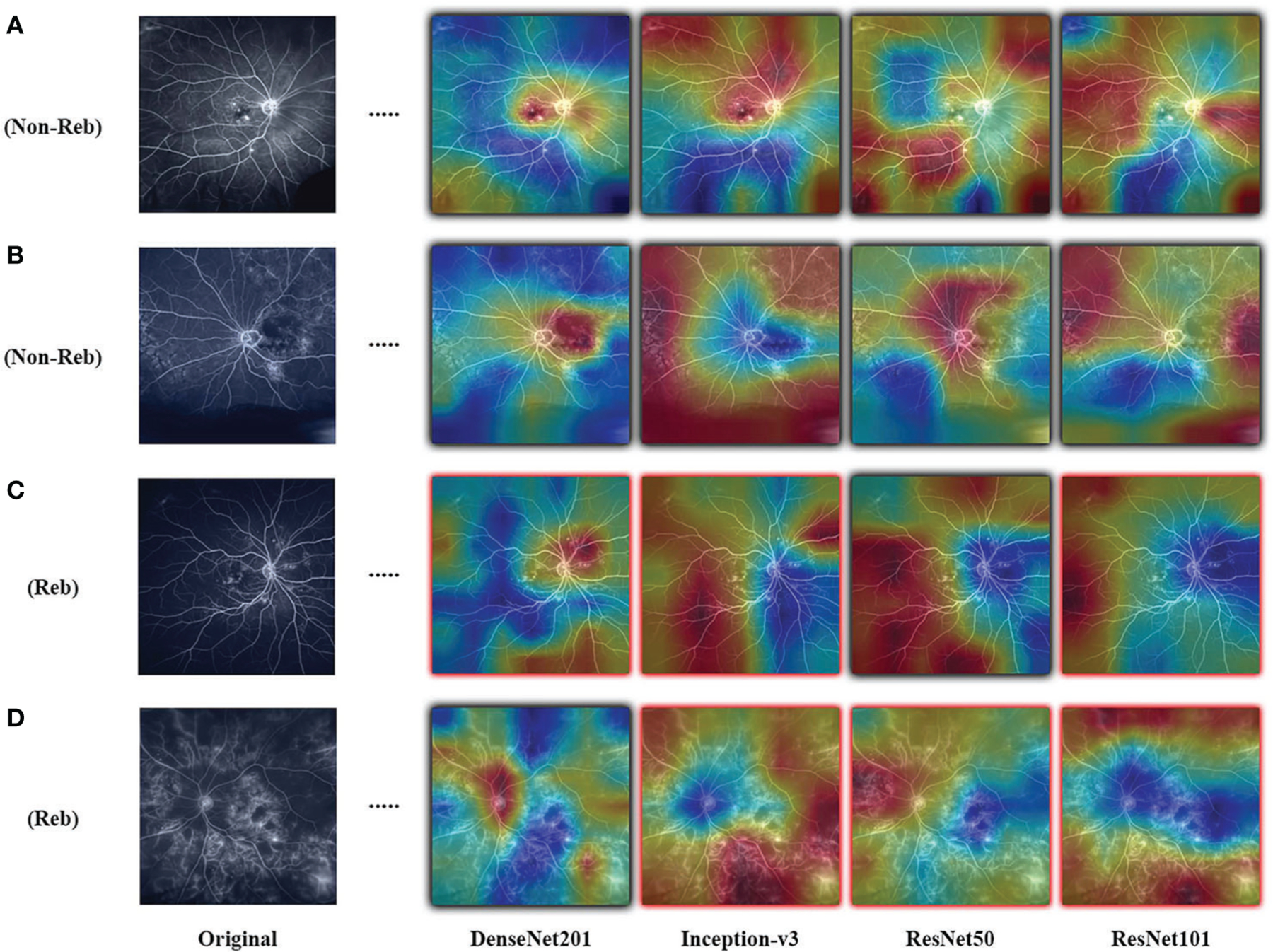
Examples of resulting heatmaps for the four trained models on rebounder and non-rebounder UWFA samples. Black outlines indicate correct classification of the sample, while red outlines indicate incorrect classification. Areas within the heatmap that are warm toned (red) represent areas where DL models have high attention and areas that are cold toned (blue) represent areas with low attention. Resulting heatmaps observed across different model architectures are inconsistent for the same sample, as attention is focused at different locations.

Results are observed for DL-based classification of UWFA late scans. The model AUC and accuracy are averaged over the 30 seeded runs of LOOCV, with the best performing model (DenseNet201) only reaching a mean AUC of 0.507 ± 0.042. The highest mean accuracy across the 30 seeded runs was only 0.511 ± 0.075. The complete results for the four model architectures are presented in Table 3, and an example of the resulting CAMs are presented in Figure 2. Confusion matrices for a single seeded run of LOOCV is show in Figure S1 of the Supplementary Material.

### 3.2 Experiment 2: utility of DL on limited OCT data

The ability of DL to predict treatment response was further evaluated by conducting experiments on the OCT imaging data of the same patient cohort from the PERMEATE dataset. Macular cube scans with 128 B-scans covering a nominal 6×6 mm scan area of 28 eyes were analyzed by a semi-automated segmentation and feature extraction software, OCTViewer (Cleveland Clinic, Cleveland, Ohio) (25). The software generated segmentation masks for both IRF and SRF objects. B-scans along with their corresponding fluid compartment masks are imported to be used with transfer learning architecture. The values specific to IRF and SRF regions are thresholded to separate the detected regions, and applied on the original 2D-OCT slice to extract detected fluid per slice. In addition to the individual fluid compartments, a mask of combined IRF and SRF regions was also generated. Slices with no detected fluid compartment are removed from their corresponding region dataset. All 28 samples with a total of 2046 slices remain for IRF compartments and combined fluid compartments. There was no SRF detected in 8 of the samples. 239 slices from 20 samples are included for SRF analysis. Slices included for fluid compartment analysis are cropped and resized to 224×224 to match input dimensions of the pre-trained DL models. Normalization is applied to the image slices to match ImageNet standards. Regions within the square image that do not contain fluid compartments are represented with zero pixel intensity, indicating that these areas are void of data. The use of data augmentations was also explored in an additional experiment detailed in Section 2.2 of the Supplementary Material.

Again, transfer learning classification experiments are employed as described in Section 2. Experiments are individually done based on the fluid compartment region; IRF, SRF, and both compartments combined. Similarly to the UWFA analysis, LOOCV is used due to the small sample size of PERMEATE. For the compartmentalized OCT analysis, a sample is considered as ≤ 128 masked B-scan slices generated during image pre-processing. For classification purposes, samples are classified as rebounder or non-rebounder through majority class voting of each of the extracted slices. Training is done with 50 epochs per training set and a learning rate of 0.0001. Then, CAMs are generated after training. Due to the OCT data being a stack of 2D image slices, the images used to generate the CAMs are chosen from the central regions of the stack of 2D slices (slices chosen randomly between 60 to 68). This allows for the highest chance of detected fluid compartment regions to be included for CAM analysis.

**FIGURE 3:**
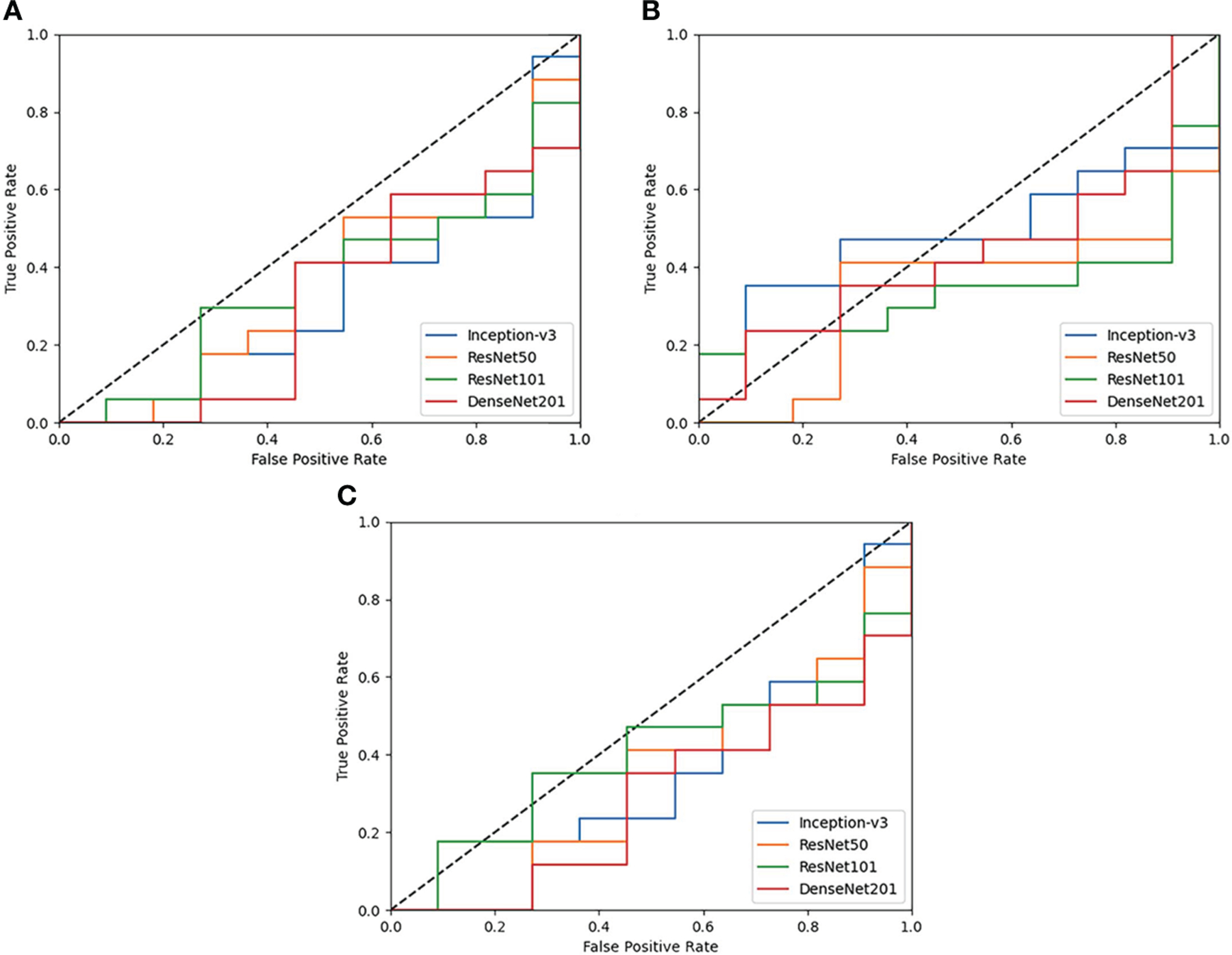
ROC plots of each tested DL model for Compartmentalized OCT experiments of **(A)** IRF regions only, **(B)** SRF regions only, and **(C)** both regions combined. Performance across the board is poor and is generally worse than random guessing (0.5 AUC shown as a dotted black line).

**FIGURE 4:**
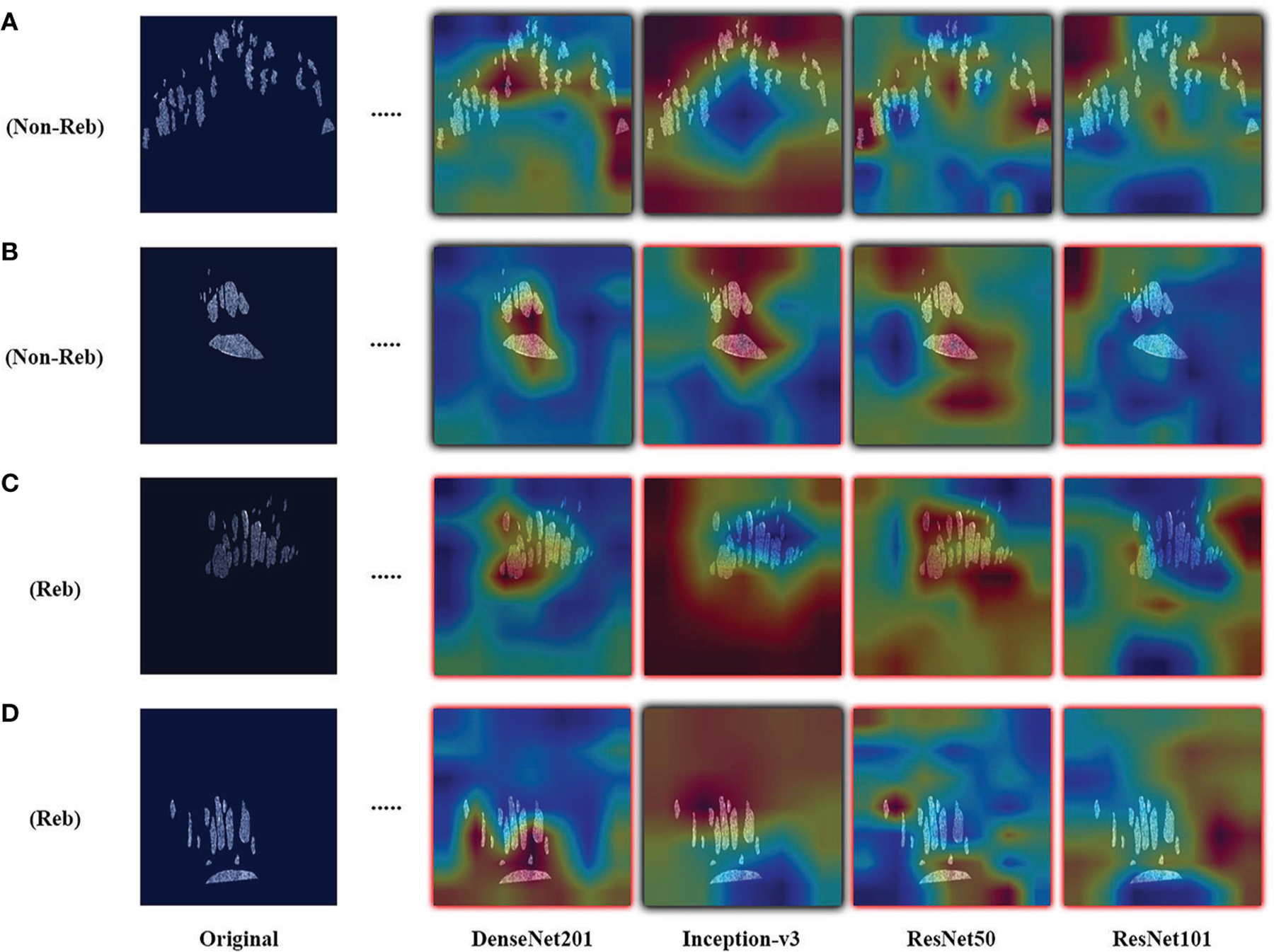
Examples of resulting heatmaps for the four trained models on rebounder and non-rebounder OCT sample slices. Samples consist of the OCT fluid compartments segmented out from IRF and SRF masks. Black outlines indicate correct classification of the sample, while red outlines indicate incorrect classification. Areas within the heatmap that are warm toned (red) represent areas where DL models have high attention and areas that are cold toned (blue) represent areas with low attention. Resulting heatmaps observed across different model architectures are inconsistent for the same sample, as attention is focused at different locations. Also, different models focused attention on regions of the image that contained no fluid compartments, suggesting that classifications were made based on areas with no fluid features at all.

Results are observed for DL-based classification of 2D compartmentalized OCT scans, grouped by fluid compartments. The best performing model architecture for classifying IRF regions was ResNet101 with a mean AUC of 0.425 ± 0.105. SRF region classification was slightly worse for each tested model architecture due to sample pruning, with the exception to DenseNet201 which performed the best with a mean AUC of 0.418 ± 0.021. The performance for combined region experiments followed similar trends to classifying IRF regions, as ResNet101 again had the best performing mean AUC of 0.443 ± 0.045. ROC curves were also generated for each fluid compartment experiment with the four tested models. Complete results can be found in Table 4, AUC plots can be found in Figure 3, and corresponding CAMs are illustrated in Figure 4. Confusionmatrices for a single seeded run of LOOCV is show in Figure S3 of the Supplementary Material.

### 3.3 Experiment 3: effect of sample size on performance

In order to evaluate the impact of sample size on DL-based classification performance, the best performing model architecture from the UWFA experiment was selected and re-trained on the Anti-VEGF dataset of increasing training set size. Five subsets were sampled from the Anti-VEGF dataset to create balanced class distributions. Image processing for these samples is the same as described for the PERMEATE UWFA experiment. Subsets increased by a factor of 28 in size from 28 to 140 eyes. This value is chosen to mirror the dataset size of PERMEATE. New instances of the transfer learning model are trained on the varying subsets and model performance is reported from 3-fold cross validation. In addition, CAMs are generated to examine how the identified ROIs change over different sample set sizes.

**FIGURE 5:**
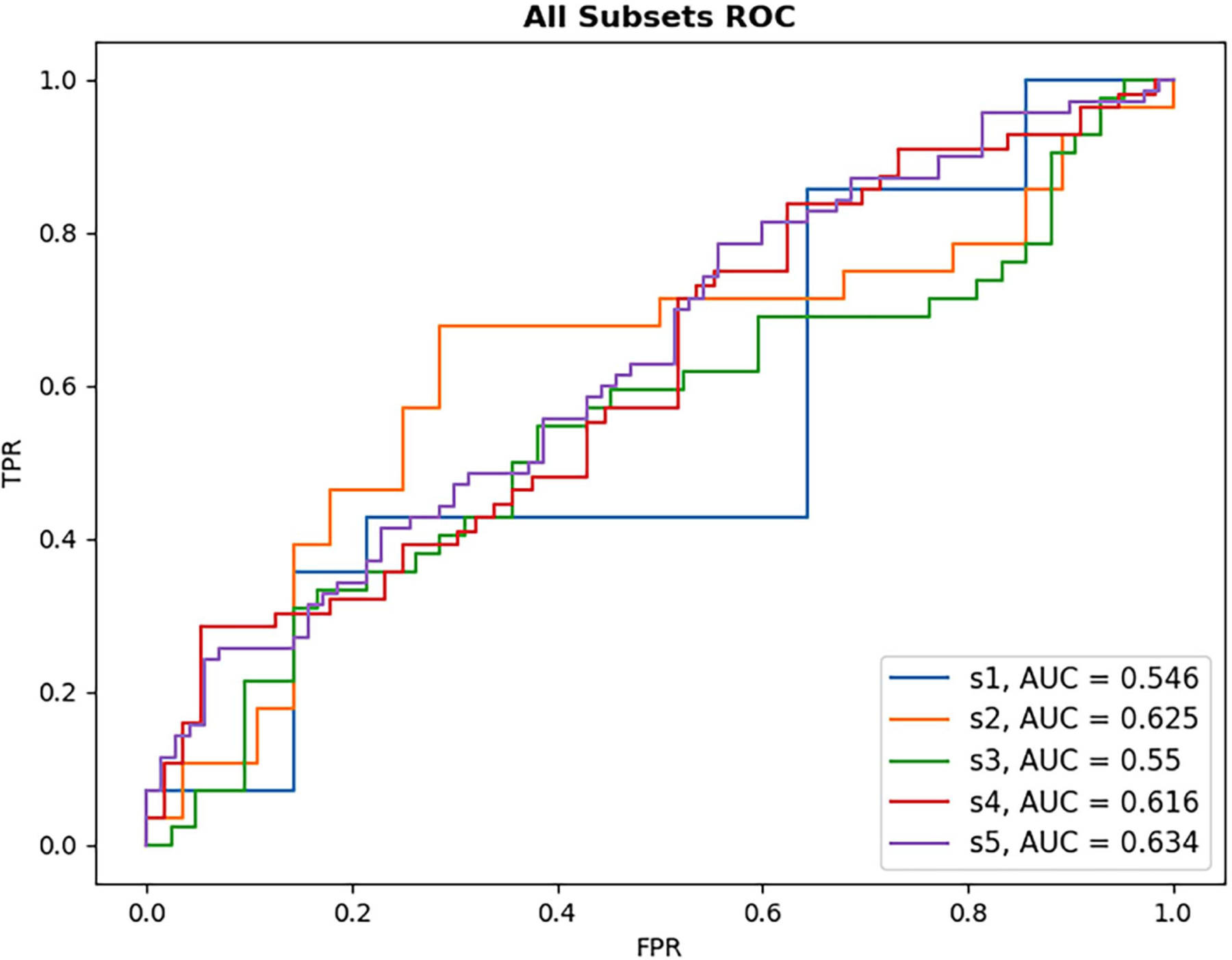
ROC plot of each DL model for anti-VEGF subset performance. Subsets increase in size with S1 being the smallest sample size and S5 the largest. As subset size increases, there is only limited observed improvement in model AUC, with the largest subset size still failing to reach high classification performance.

**FIGURE 6:**
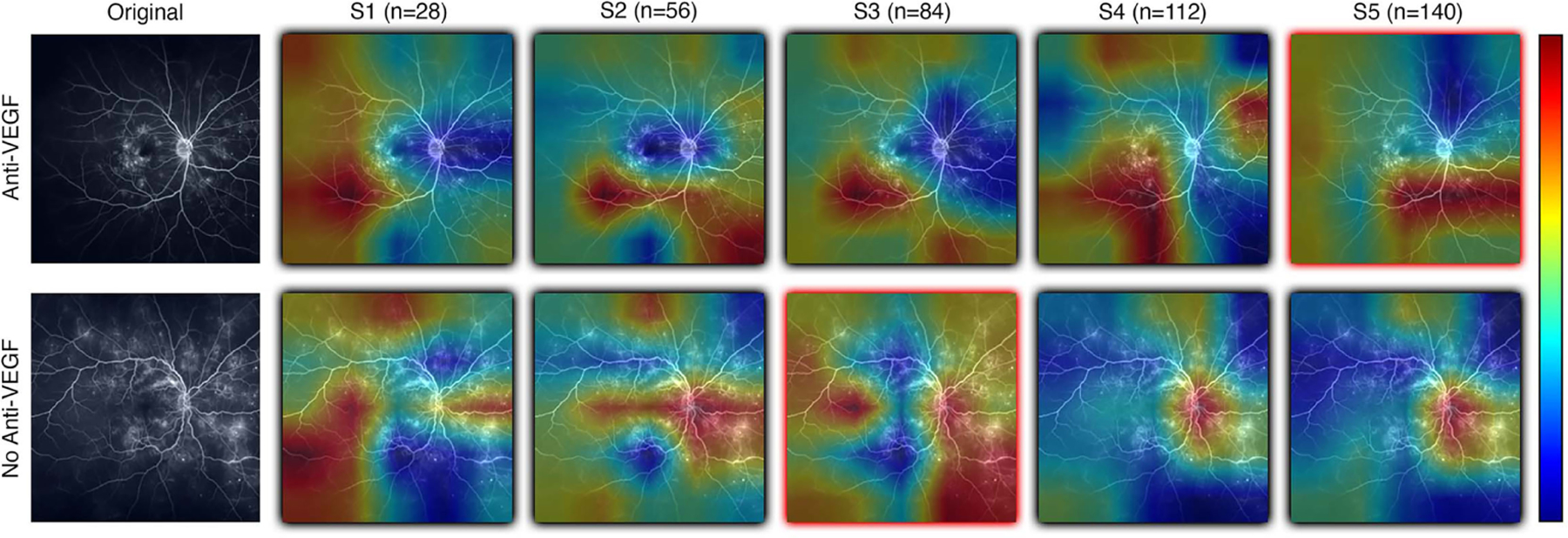
Examples of resulting heatmaps from a new instance of a trained model as subset size increases for both rebounder and non-rebounder patients. Black outlines indicate correct classification of the sample, while red outlines indicate incorrect classifications. Areas within the heatmap that are warm toned (red) represent areas where DL models have high attention and areas that are cold toned (blue) represent areas with low attention. Identified regions of interest in heatmaps vary and are inconsistent as subset size increases, indicating that DL models fail to consistently learn relevant features needed for classification.

Results from DL-based classification of the Anti-VEGF dataset are calculated from the pooled predictions over 3-fold cross validation per subset. The best performing model architecture from the PERMEATE UWFA experiment (DenseNet201) was chosen to examine the effect of sample size on performance. Subsets are denoted as S1, S2, up to S5, in order of increasing subset size. Subset 5 (largest training set size) had the best reported AUC of 0.634. These results are shown in Table 5. In addition, receiver operating characteristic (ROC) curves are generated for each subset and plotted on the same axis to show how performance changes as the subset size increases. This plot is illustrated in Figure 5. The resulting CAMs are illustrated in Figure 6.

## 4 Discussion

The objective of this study was to evaluate the utility of deep learning (DL) to predict response to treatment or future treatment need, a DL task that lacks well-defined visual indicators and large readily available datasets. The goal of the PERMEATE Ultra-widefield fluorescein angiography (UWFA) experiment was to gauge the ability for DL to classify samples as rebounders and non-rebounders with the PERMEATE dataset, where training set size was small. Even when utilizing transfer learning, a DL technique that is tailored towards image classification problems, performance is sub-optimal regardless of model architecture. Over 30 averaged runs, both the average AUC and accuracy are at or slightly less than random chance, indicating that for this more challenging DL task of predicting treatment response, DL has failed to converge to an accurate representation of the problem. Furthermore, upon analysis of resulting class activation maps (CAMs), it is clear that the DL models are inconsistent in identifying regions of interest (ROI) that lead to classification of the sample. Identified ROI are sporadic, and alternate between focusing on the optic nerve, macula, or peripheral regions. Additionally, fluid leakage did not seem to consistently be identified as ROI by the model. These results suggest that for the UWFA modality, DL is unable to detect consistent visual indicators from the limited training set size related to predicting therapeutic durability in this disease set.

Similar to the UWFA analysis, the goal of the PERMEATE Optical coherence tomography (OCT) experiment was to observe DL performance on a small dataset but with compartmentalized OCT data. Similarly, the transfer learning models failed to reach optimal performance in predicting therapeutic durability. In fact, for the compartmentalized OCT analysis, the transfer learning models failed to even consistently reach random chance. Subretinal fluid (SRF) classification was exceptionally poor, most likely due to the limited number of samples as 2D slices that contained detected SRF were minimal (n=10). Furthermore, the combination of intraretinal fluid (IRF) and SRF was subject to increased bias due to the small number of SRF samples. Similar trends are observed for the resulting CAMs for the compartmentalized OCT images. Identified areas of attention are again very inconsistent for the four different model architectures. As a result of attempting DL classification for two modalities of the PERMEATE dataset, it can be concluded that DL was unable to derive accurate representations for this particular treatment prediction task.

Given the results of DL classification on the PERMEATE dataset, it was important to also determine if the issue with the sub-optimal classification performance was due to the training set size, or the task itself. The task of classifying eyes based on tolerance of treatment interval is much more complex than classifying based on need for anti-vascular endothelial growth factor (Anti-VEGF) therapy. From the results of the UWFA subset experiment on the Anti-VEGF dataset, it was observed that there was only a marginal improvement in DL performance as the training set size increased for UWFA images. Even when the training set size increased by a magnitude of five (though still a relatively small dataset), DL performance was only slightly better than random chance. Thus, DL appears to fail for this task of predicting treatment response due to the inability to learn necessary features and representations from UWFA data with concurrent data scarcity. However, treatment response imaging datasets for eyes afflicted with diabetic eye disease that are large in sample size are also limited, which will lead to even worse DL classification performance, particularly in UWFA data. This further suggests that DL might not be optimally suited for such a task, and other radiomic-based feature-based approaches should be considered as an alternative.

Such approaches include hand-crafted or radiomic feature extraction techniques. Radiomics is a technique of extracting and analyzing quantitative image features from medical images with image processing techniques, with the goal of identifying correlations between biologically relevant features to support clinical decision making (26). Previous studies have shown the efficacy of handcrafted feature-based approaches (like radiomics) in predicting treatment response from UWFA images, where high predictive performance is reported on the PERMEATE dataset, a result that was not reproducible with DL. Prasanna et al. presented two novel UWFA-derived radiomics biomarkers from the PERMEATE study. The first being a biomarker that captures the differences in spatial arrangement of leakage patterns for eyes more tolerant of extended interval dosing and those that do not. The second biomarker relates to the variance of vessel tortuosity between eyes with different levels of tolerance to treatment (13). These feature extraction-based approaches are better suited for this task, as even with limited data, radiomics was able to identify FA-derived leakage morphology and vessel tortuosity-based biomarkers that discriminated eyes that were rebounders and non-rebounders to treatment with a cross-validated area under the receiver operating characteristic curve (AUC) of 0.77 ± 0.14 for baseline leakage distribution features and 0.73 ± 0.10 for UWFA baseline tortuosity measures (13, 14). Additionally, a set of texture-based radiomics features were extracted from each of the fluid and retinal tissue compartments of the PERMEATE OCT images and yielded a cross-validated AUC of 0.78 ± 0.08 for distinguishing rebounders from non-rebounders (27). These findings suggest that algorithms attempting to identify pre-defined patterns in the image might require fewer training images compared to unsupervised feature generation approaches such as DL.

Recent works have also evaluated the utility of DL for predicting treatment tolerance of eyes with various ocular diseases, such as diabetic macular edema (DME) (28–30). Rasti et al. achieved an average AUC of 0.855 in discriminating rebounders from non-rebounders using DL on a retrospective study of 127 subjects (28). Outside of DME, Feng et al. demonstrated that predictive AUC can reach levels greater than 0.80 for predicting effectiveness of therapy for choroidal neovascularization (CNV) and cystoid macular edema (CME) afflicted eyes in a study containing 228 patients (29). Finally, using a dataset that included 183,402 OCT B-scans, Prahs et al. developed a DL model capable of distinguishing OCT B-scans that require intravitreal injection from those that do not, yielding above 95% classification accuracy on a validation dataset (30). One distinction for this study is that it instead predicted need for treatment as opposed to tolerance to treatment. These recent studies indicate that there is promise in DL-based algorithms for ocular related tasks, when provided with a large enough dataset.

We do acknowledge that this study has important limitations. The major limitation revolves around the extremely limited dataset size (n=28). The small dataset size causes DL models to be prone to overfitting, as well as limits the possible cross validation techniques used. That said, the primary goal of this analysis was to evaluate the potential for DL in a data scarce environment, particularly in a dataset that has previously been able to be evaluated with other image feature characterization techniques. The compartmentalized approach to OCT DL assessment also has an important limitation of cross-study comparison, as most other OCT DL analyses utilized full image or region-of-interest assessments for their studies. An additional important limitation is related to the specifics of defining treatment response/durability, as well as the need for anti-VEGF treatment. These are challenging definitions based on variable perspectives on timing, what qualifies as a failure, and variable physician-based decisions on need for therapy. The *post-hoc* retrospective nature of the analysis creates challenges specifically around these definitions. A future prospective comparative assessment of DL versus hand-crafted feature characterization or even hybrid techniques would be of high value to consider for additional validation. Furthermore, a separate independent validation dataset would alleviate this issue by providing an avenue for model validation that did not limit the training size.

## 5 Conclusion

In spite of the aforementioned limitations, the present study was able to evaluate the limitations of DL for predicting anti-VEGF treatment response across variable imaging modalities and sample sizes. The findings of this study have allowed for potentially identifying the circumstances where deep learning fails, mainly when given a more complex task for which data is not as readily available. Even after an exhaustive search of model architectures and model hyperparameters, performance markedly better than random guessing was unobtainable. This was further supported by resulting class activation maps that showed the lack of consistent identification of regions of interest that were learned by the deep learning models. The findings in this study appear to suggest that deep learning is not the blanket approach for predicting therapeutic response in ophthalmology, and other approaches should possibly be evaluated and considered in parallel.

## Supplementary Material

PERMEATE_Supplementary_Material

## Figures and Tables

**TABLE 1:** PERMEATE patient characteristics.

Characteristic		Mean	Std Dev	Count	Column N %
Age		67.17	9.71		
Central Subfield Thickness		541.17	246.59		
Letter Score		54.34	23.34		
Macular Volume		13.70	4.30		
Diagnosis	DME			14	48.30%
	RVO			15	51.70%
	CRVO			10	34.50%
	BRVO			5	17.20%
Gender	Female			18	62.10%
	Male			11	37.90%
Glaucoma	No			20	69.00%
	Yes			9	31.00%
Hypertension	No			6	20.70%
	Yes			23	79.30%
Lens Status	Phakic			22	75.90%
	Pseudophakic			7	24.10%
Race	African-American			6	20.70%
	Caucasian			23	79.30%
Study Eye	Left (OS)			12	41.40%
	Right (OD)			17	58.60%
VA Worse at V8	No			17	58.60%
	Yes			12	41.40%

**TABLE 2:** Anti-VEGF patient characteristics.

Characteristic		Mean	Std Dev	Count	Column N %
Age		61.83	13.12		
Clinical DME	No			125	57.60%
	Yes			92	42.40%
Diagnosis	Mild NPDR			19	8.76%
	Moderate NPDR			53	24.42%
	Severe NPDR			92	42.40%
	PDR			53	24.42%
Gender	Female			96	44.24%
	Male			121	55.76%
Hypertension	No			10	4.61%
	Yes			207	95.39%
Lens Status	Phakic			160	73.73%
	Pseudophakic			57	26.27%
Race	African-American			99	45.62%
	Asian			1	0.46%
	Caucasian			109	50.23%
	Multiracial/Multicultural			1	0.46%
	Unavailable			7	3.23%
Study Eye	Left (OS)			87	40.09%
	Right (OD)			130	59.91%
Required Anti-VEGF	No			76	35.02%
Treatment	Yes			141	64.98%

**TABLE 3:** PERMEATE UWFA Results (over 30 runs).

Metric	ResNet50	ResNet101	Inception-v3	DenseNet201
Mean AUC	0.491 ± 0.036	0.504 ± 0.032	0.487 ± 0.076	0.507 ± 0.042
Mean ACC	0.511 ± 0.075	0.468 ± 0.072	0.488 ± 0.095	0.508 ± 0.082

**TABLE 4:** PERMEATE OCT AUC results (over 5 runs).

OCT Compartments	ResNet50	ResNet101	Inception-v3	DenseNet201
IRF Regions	0.414 ± 0.053	0.425 ± 0.105	0.407 ± 0.034	0.386 ± 0.055
SRF Regions	0.367 ± 0.026	0.347 ± 0.030	0.400 ± 0.052	0.418 ± 0.021
Combined Regions	0.399 ± 0.056	0.443 ± 0.045	0.409 ± 0.060	0.379 ± 0.063

**TABLE 5:** Anti-VEGF UWFA results (3-fold cross validation).

	S_1_	S_2_	S_3_	S_4_	S_5_
Pooled AUC	0.546	0.625	0.550	0.616	0.634

## Data Availability

The original contributions presented in the study are included in the article/Supplementary Material. Further inquiries can be directed to the corresponding authors.
