# Supplementary material for: Evaluating the utility of deep learning for predicting therapeutic response in diabetic eye disease
Source: Front Ophthalmol (Lausanne). Author manuscript; Available in PMC 2023 Feb 2. (PMC9894083; doi:10.3389/fopht.2022.852107)
Supplement: PERMEATE_Supplementary_Material [file NIHMS1861389-supplement-PERMEATE_Supplementary_Material.pdf]

Supplementary Material

1 EFFECT OF DATA AUGMENTATIONS ON PERFORMANCE

Experiments 1 (Utility of DL on Limited UWFA data) and 2 (Utility of DL on Limited OCT data) were repeated with additional data augmentations. Due to differences in the representation of the two imaging modalities, data augmentations were applied specifically to the imaging modality analyzed.

1.1 Effect of Data Augmentations on Limited UWFA data

The ability for DL to predict treatment response on UWFA imaging data was evaluated on the PERMEATE dataset in a similar manner to Experiment 1. In addition to the original image preprocessing (resizing and center crop), random augmentations such as rotations, horizontal, and vertical flips were applied to the UWFA images with random probabilities. These data augmentations artificially increase the amount of training data provided to the DL models, somewhat addressing the extreme limited dataset size (n=29) of PERMEATE.

The training process remains the same as the original experiment. Transfer learning is used with the four model architectures (ResNet50, ResNet101, Inception-v3, DenseNet201) and leave-one-out cross validation (LOOCV) is employed to maximize training set sizes.

Model AUC and accuracy are averaged over the same 30 seeded runs of LOOCV used in Experiment 1 to compare the effect of data augmentations on resulting performance. The complete results for the four model architectures are presented in Table S1.

Table S1. PERMEATE Data Augmentation UWFA Results (over 30 runs)

Metric	ResNet50	ResNet101	Inception-v3	DenseNet201
Mean AUC	0.508 ± 0.040	0.505 ± 0.036	0.485 ± 0.037	0.515 ± 0.053
Mean ACC	0.523 ± 0.035	0.466 ± 0.068	0.494 ± 0.072	0.506 ± 0.086

1.2 Effect of Data Augmentations on Limited OCT data

The ability for DL to predict treatment response on OCT imaging data was evaluated on the PERMEATE dataset in a similar manner to Experiment 2. The same additional data augmentations used for the UWFA experiments were also utilized for OCT data. However, the random augmentations were applied at a sample-level and not at the individual OCT B-scan slice level.

Again, the training process remains the same as the original experiment. Transfer learning is used with each of the four model architectures with LOOCV. Fluid compartments are segmented and applied as masks to each OCT B-scan. Slices that are void of pixel data are pruned from the sample, such that ≤ 128 masked B-scan slices make up one sample. Samples are classified based on majority class voting over each predicted slice.

Model AUC is reported as an average over the same five seeded runs of LOOCV used in Experiment 2. Results are reported per individual fluid compartment, and also through a combined region analysis. The complete results of the four model architectures for each region analysis is shown in Table S2.

Table S2. PERMEATE OCT AUC Results (over 5 runs)

OCT Compartments	ResNet50	ResNet101	Inception-v3	DenseNet201
IRF Regions	0.424 ± 0.045	0.447 ± 0.074	0.411 ± 0.038	0.396 ± 0.055
SRF Regions	0.364 ± 0.021	0.358 ± 0.030	0.400 ± 0.028	0.380 ± 0.016
Combined Regions	0.406 ± 0.053	0.458 ± 0.052	0.423 ± 0.033	0.415 ± 0.037

2 CONFUSION MATRICES

Confusion matrices for experiments conducted on the PERMEATE dataset are shown for each specific experiment per model architecture, with and without data augmentations.

2.1 UWFA Confusion Matrices

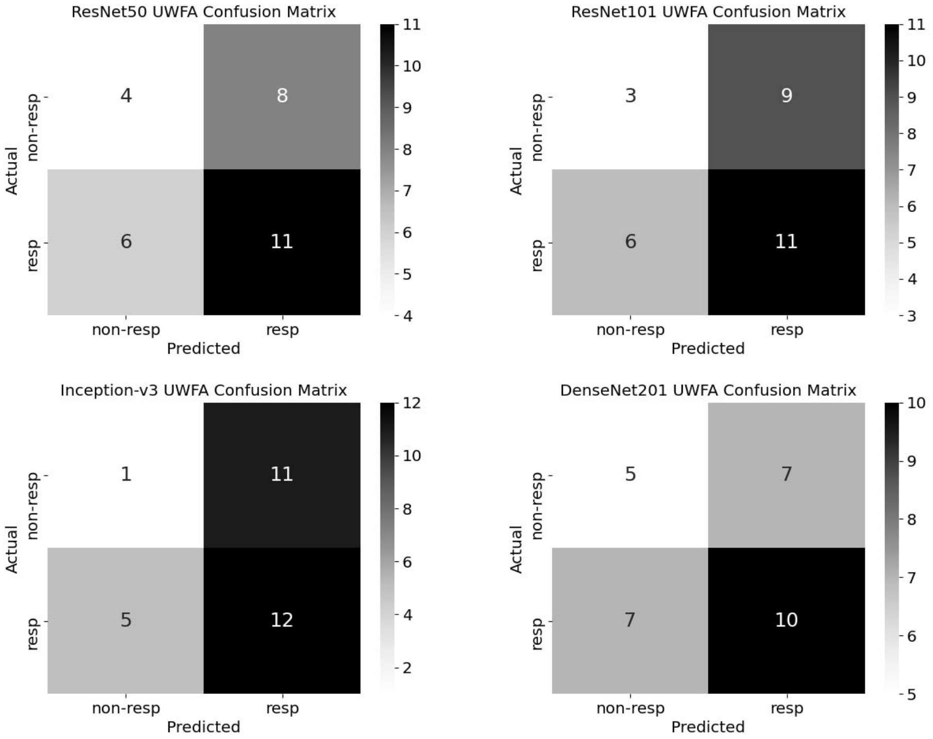

Figure S1. Confusion matrices for each of the four tested model architectures trained on PERMEATE UWFA data with no data augmentations.

2.2 OCT Confusion Matrices

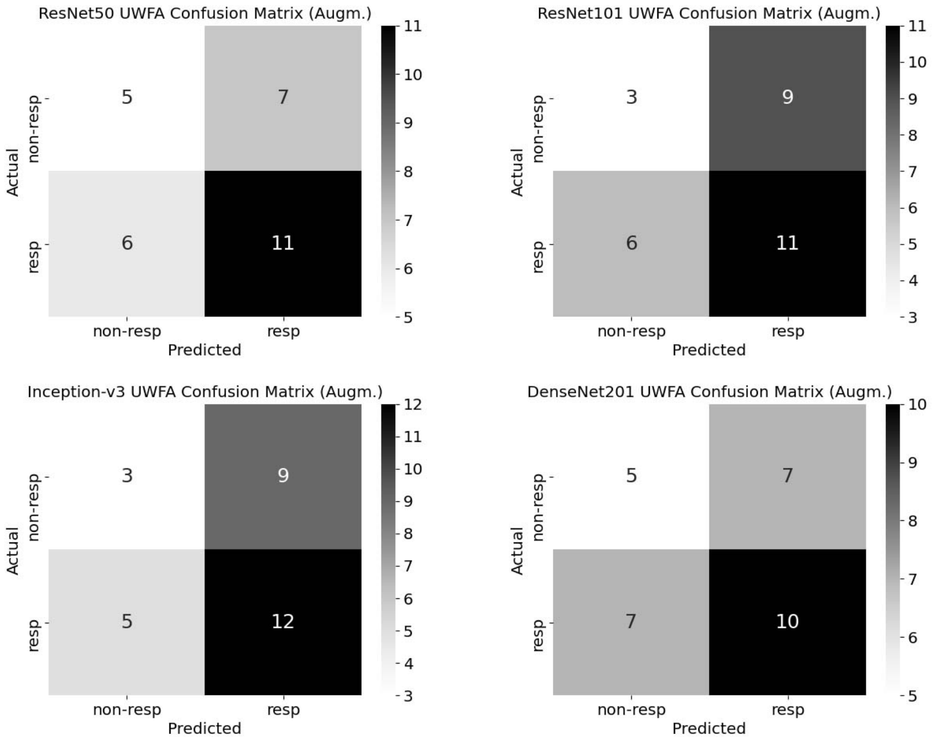

Figure S2. Confusion matrices for each of the four tested model architectures trained on PERMEATE UWFA data with data augmentations.

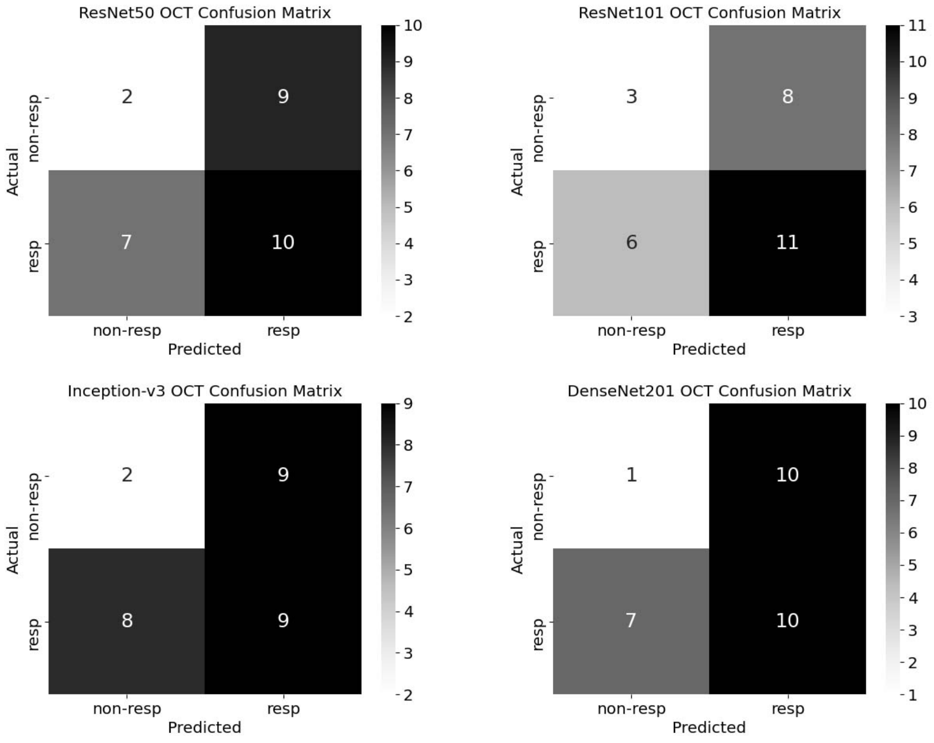

Figure S3. Confusion matrices for each of the four tested model architectures trained on PERMEATE OCT data with no data augmentations. Both segmented IRF and SRF regions are combined for analysis.

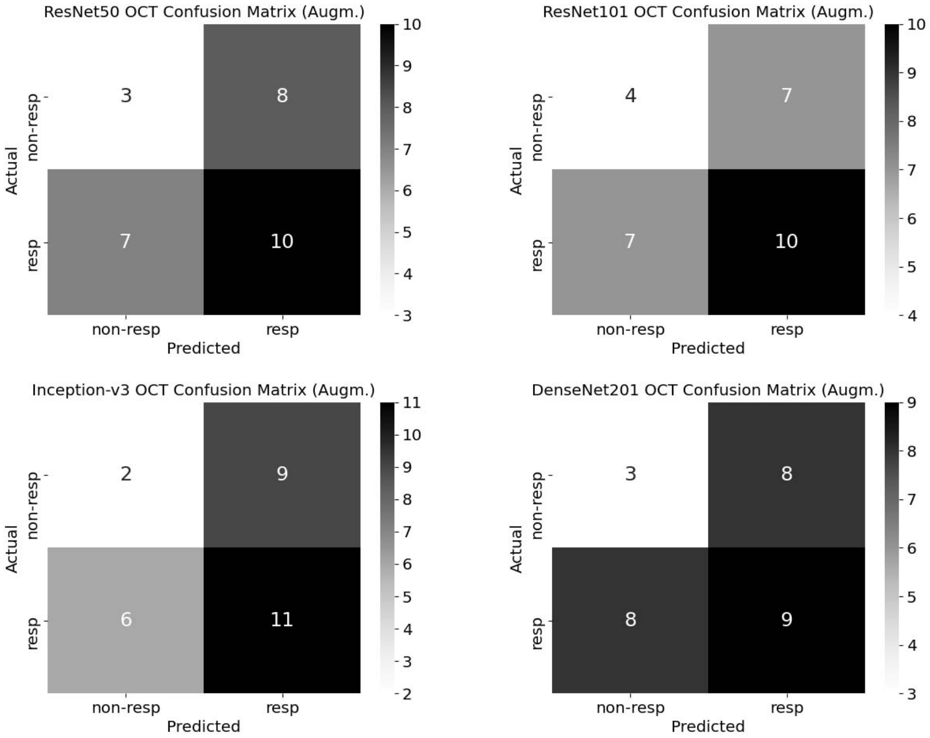

Figure S4. Confusion matrices for each of the four tested model architectures trained on PERMEATE OCT data with data augmentations. Both segmented IRF and SRF regions are combined for analysis.
