# Supplementary material for: Evaluating the utility of deep learning for predicting therapeutic response in diabetic eye disease
Source: Front Ophthalmol (Lausanne). 2022 Aug 12;2:852107. doi: 10.3389/fopht.2022.852107 (PMC9894083; doi:10.3389/fopht.2022.852107)
Supplement: Supplementary file 1 [file DataSheet_1.pdf]

Supplementary Material

1 EFFECT OF PATCH-BASED IMAGE PRE-PROCESSING ON LIMITED UWFA DATA

Experiment 1 (Utility of DL on Limited UWFA data) was repeated with different image pre-processing steps to investigate the utility of DL on limited UWFA data with a patch-based approach.

Instead of aggressively down-sampling the image from 3900x3072 pixels to 224x224 pixels, the UWFA samples are instead center cropped to 2912x2912 pixels and then non-overlapping patches are extracted. A total of 196 patches are extracted from each image. To remove artifacts from the outer regions of the image, only the 25 center patches are included for each sample. All other image pre-processing steps remained the same as described in Experiment 1.

The training process has one modification due to the introduction of image patches. Each sample comprises 25 patches, and all patches are utilized in the training and evaluation of the DL models. Each patch is assigned a prediction from the trained DL model, and the final classification of the evaluation sample is determined through majority class voting. Transfer learning is used with four different model architectures (ResNet50, ResNet101, Inception-v3, DenseNet201) and leave-one-out cross-validation (LOOCV) to maximize training set sizes.

Model AUC and accuracy are averaged over the same 30 seeded runs of LOOCV used in Experiment 1, performed to compare the effects of a patch-based approach on resulting performance. The complete results for the four model architectures are presented in Table S1.

Table S1. PERMEATE Patch-Based UWFA Results (over 30 runs)

Metric	ResNet50	ResNet101	Inception-v3	DenseNet201
Mean AUC	0.487 ± 0.032	0.498 ± 0.035	0.494 ± 0.060	0.487 ± 0.051
Mean ACC	0.449 ± 0.040	0.456 ± 0.020	0.444 ± 0.031	0.460 ± 0.041

The performance of the transfer learning models decreased when using a patch-based approach, compared to the performance of the same models trained on the whole image. For example, when trained on the whole image, the best-performing model (DenseNet201) had a mean AUC of 0.507 ± 0.042 but when trained with a patch-based approach, DenseNet201 had a lower mean AUC of 0.487 ± 0.051. Similar trends were noted for the other model architectures. These results are unsurprisingly given the limited dataset size. In this experiment of extracting patches from the full-size UWFA image, many seeds resulted in the trained model predicting a single class (all rebounders or all non-rebounders). Overall, the results of this supplemental experiment highlight the association between limited dataset size to poor classification performance of DL models for this study.

2 EFFECT OF DATA AUGMENTATIONS ON PERFORMANCE

Experiments 1 (Utility of DL on Limited UWFA data) and 2 (Utility of DL on Limited OCT data) were repeated with additional data augmentations. Due to differences in the representation of the two imaging modalities, data augmentations were applied specifically to the imaging modality analyzed.

2.1 Effect of Data Augmentations on Limited UWFA data

The ability of DL to predict treatment response on UWFA imaging data was evaluated on the PERMEATE dataset in a similar manner to Experiment 1. In addition to the original image preprocessing (resizing and center crop), random augmentations such as rotations, horizontal, and vertical flips were applied to the UWFA images with random probabilities. Using PyTorch's data augmentation suite, each image in the training set is rotated at a random angle. Then, the images are flipped horizontally with a probability of 0.5 and flipped vertically with a probability of 0.5. For one run, each training sample is randomly augmented for each of the 29 iterations of LOOCV. This is repeated for all 30 runs. These data augmentations artificially increase the amount of training data provided to the DL models, somewhat addressing the extremely limited dataset size (n=29) of PERMEATE.

The training process remains the same as the original experiment of evaluating the performance of DL to predict anti-VEGF treatment response on UWFA imaging from the PERMEATE dataset. Transfer learning is used with the four model architectures (ResNet50, ResNet101, Inception-v3, DenseNet201) and leave-one-out cross-validation (LOOCV) is employed to maximize training set sizes, where through each iteration of LOOCV, one sample is set aside and used for model validation and the rest of the samples are used for training. This is repeated for all configurations of the dataset where a sample is used as hold-out validation.

Model AUC and accuracy are averaged over the same 30 seeded runs of LOOCV used in Experiment 1 to compare the effect of data augmentations on resulting performance. The complete results for the four model architectures are presented in Table S2.

Table S2. PERMEATE Data Augmentation UWFA Results (over 30 runs)

Metric	ResNet50	ResNet101	Inception-v3	DenseNet201
Mean AUC	0.508 ± 0.040	0.505 ± 0.036	0.485 ± 0.037	0.515 ± 0.053
Mean ACC	0.523 ± 0.035	0.466 ± 0.068	0.494 ± 0.072	0.506 ± 0.086

Introducing data augmentations had a marginal improvement in classification performance over UWFA data. However, noted improvements were not substantial enough to result in optimal classification performance. For example, when trained with no data augmentation, the best-performing model (DenseNet201) had a mean AUC of 0.507 ± 0.042. With data augmentation, DenseNet201 achieved a mean AUC of 0.515 ± 0.053, which is an improvement, but still not a result that can be considered optimal for this task.

2.2 Effect of Data Augmentations on Limited OCT data

The ability of DL to predict treatment response on OCT imaging data was evaluated on the PERMEATE dataset in a similar manner to Experiment 2. The same additional data augmentations used for the UWFA experiments were also utilized for OCT data, where each sample in the training set is subjected to an image rotation by a random degree, and horizontal flips and vertical flips with a probability of 0.5. However, the random augmentations were applied at a sample level and not at the individual OCT B-scan slice level. Again, these augmentations are applied per training sample for each iteration of LOOCV, where one of the 28 OCT samples is set aside as held-out validation. This process is repeated over 5 randomly seeded runs.

Again, the training process remains the same as the original experiment of evaluating DL performance for predicting anti-VEGF treatment response on OCT imaging from the PERMEATE dataset. Transfer learning is used with each of the four model architectures with LOOCV. Fluid compartments are segmented and applied as masks to each OCT B-scan. Slices that are void of pixel data are pruned from the sample, such that ≤ 128 masked B-scan slices make up one sample. Samples are classified based on majority class voting over each predicted slice.

Model AUC is reported as an average over the same five seeded runs of LOOCV used in Experiment 2. Results are reported per individual fluid compartment, and also through a combined region analysis. The complete results of the four model architectures for each region analysis are shown in Table S3.

Table S3. PERMEATE OCT AUC Results (over 5 runs)

OCT Compartments	ResNet50	ResNet101	Inception-v3	DenseNet201
IRF Regions	0.424 ± 0.045	0.447 ± 0.074	0.411 ± 0.038	0.396 ± 0.055
SRF Regions	0.364 ± 0.021	0.358 ± 0.030	0.400 ± 0.028	0.380 ± 0.016
Combined Regions	0.406 ± 0.053	0.458 ± 0.052	0.423 ± 0.033	0.415 ± 0.037

Data augmentations again had a slight improvement in classification performance on the OCT imaging modality. Again, the noted improvements as a result of introducing data augmentations did not result in optimal model classification performance. For example, the best-performing model for classifying IRF regions without data augmentations was ResNet101, which achieved a mean AUC of 0.425 ± 0.105. The same model architecture improved performance to a mean AUC of 0.447 ± 0.074 when introducing data augmentations, but this result is still suboptimal (still like random guessing). The best performing model for classifying SRF regions without data augmentations was DenseNet201, which had a reported mean AUC of 0.418 ± 0.021. However, when introducing augmentations, DenseNet201 had a lower mean AUC of 0.380 $\pm$ 0.016. As noted in the original experiment, this is likely due to the small distribution of samples that contained detected SRF compartments, further exacerbating the problem of limited data. Finally, the best performing model for classifying the combined regions without data augmentations was ResNet101, which had a mean AUC of 0.443 ± 0.045. Again, with the inclusion of data augmentations, ResNet101 achieved slightly better results, with a reported AUC of 0.458 ± 0.052. Overall, while data augmentations boosted performance marginally, it still did not allow for the model architectures to achieve optimal classification performance.

3 CONFUSION MATRICES

Confusion matrices for experiments conducted on the PERMEATE dataset are shown for each specific experiment per model architecture, with and without data augmentations.

3.1 UWFA Confusion Matrices

Confusion matrices for experiments on UWFA data are shown in Figure S1 and Figure S2.

3.2 OCT Confusion Matrices

Confusion matrices for experiments on OCT data are shown in Figure S3 and Figure S4.

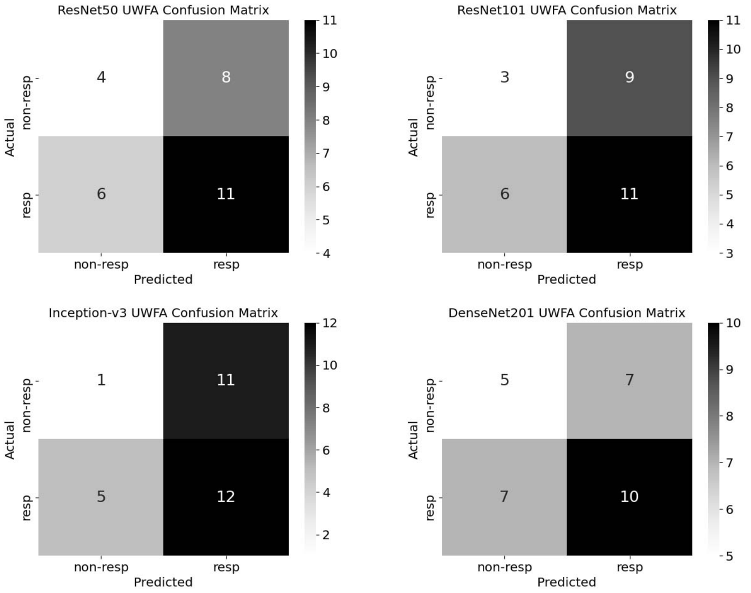

Figure S1. Confusion matrices for each of the four tested model architectures trained on PERMEATE UWFA data with no data augmentations. The confusion matrices indicate a preference for the DL models to predict a sample as a responder for anti-VEGF therapy. This bias is relatively consistent across the four model architectures. However, it should be noted that the model does not only predict the responder class, and still attempts to classify samples as non-responders. Overall, all trained models had a very high false-positive rate.

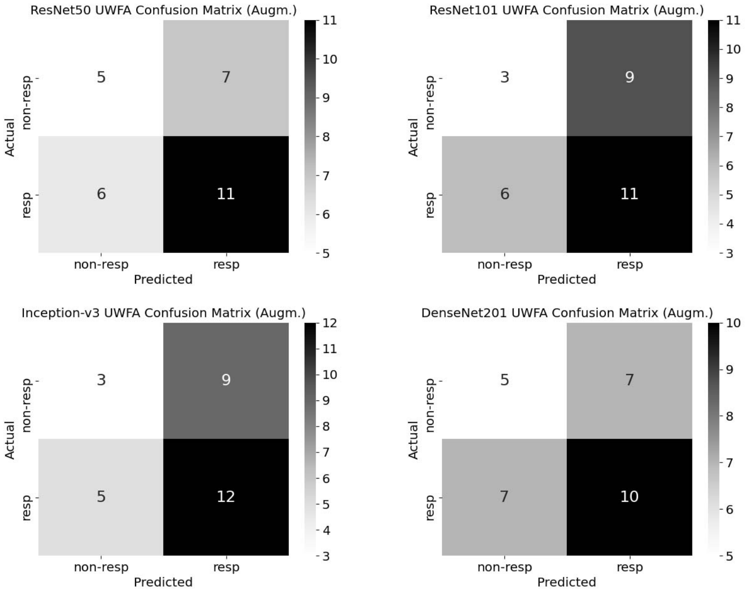

Figure S2. Confusion matrices for each of the four tested model architectures trained on PERMEATE UWFA data with data augmentations. The confusion matrices again indicate a preference for the DL models to predict a sample as a responder for anti-VEGF therapy. This bias is relatively consistent across the four model architectures, and it also appears that the inclusion of data augmentations did not drastically change this preference to predict the responder class. However, it should be noted that the model does not only predict the responder class, and indeed still attempts to classify samples as non-responders. Overall, all trained models had a very high false-positive rate.

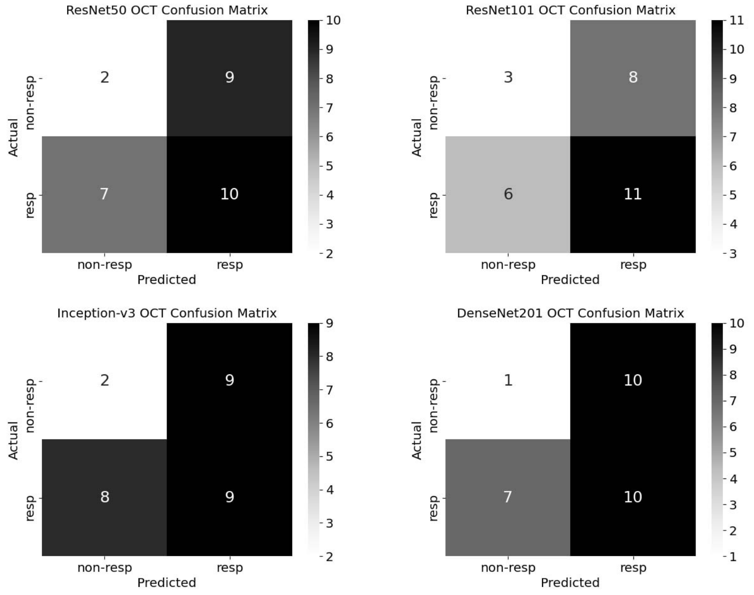

Figure S3. Confusion matrices for each of the four tested model architectures trained on PERMEATE OCT data with no data augmentations. Both segmented IRF and SRF regions are combined for analysis. A similar trend of misclassifications is observed. The four model architectures had trouble in correctly classifying non-responder samples, with a bias of incorrectly predicting these samples as responders. There also was worse a much higher false-negative rate than observed in the UWFA experiments. Overall, the DL models struggled in classifying the OCT samples, with both high false-positive and false-negative rates.

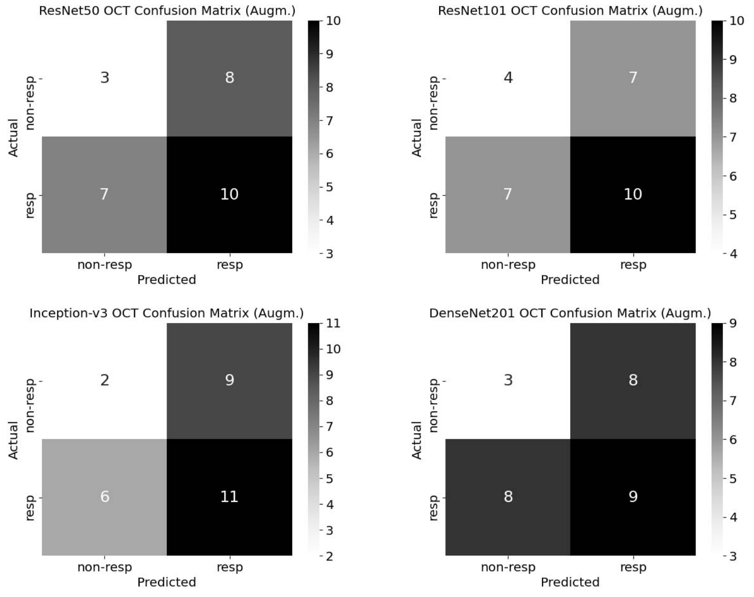

Figure S4. Confusion matrices for each of the four tested model architectures trained on PERMEATE OCT data with data augmentations. Both segmented IRF and SRF regions are combined for analysis. Even with added augmentations, the confusion matrices again showcase a similar trend of having trouble in classifying non-responder samples. The DL models struggled in classifying the OCT samples, with both high false-positive and false-negative rates.
